# Opioidrotation bei „mixed pain“ in der Tumorschmerztherapie: Wie aus einer „chronischen Schmerzpatientin“ eine „Palliativpatientin“ wird – ein Fallbericht

**DOI:** 10.1007/s10354-021-00889-9

**Published:** 2021-10-01

**Authors:** Magdalena Demuth

**Affiliations:** 1 Palliativeinrichtung, Krankenhaus der Elisabethinen Graz GmbH, Elisabethinergasse 14, 8020 Graz, Österreich; 2 Paracelsus Medical University, Strubergasse 21, 5020, Salzburg, Österreich

**Keywords:** Neuropathischer Schmerz, Tumorschmerz, Gemischter Schmerz, Opioidrotation, Palliativmedizin, Oxycodon, Neuropathic pain, Cancer pain, Mixed pain, Opioid switching, Palliative care, Oxycodon

## Abstract

Im vorliegenden Fallbericht wird die Situation einer 67-jährigen chronischen Schmerzpatientin geschildert, die aufgrund eines Zufallsbefundes mit der Diagnose eines metastasierten kleinzelligen Bronchialkarzinoms konfrontiert wird. Bisher hatte sie mit einer chronischen Lumboischialgie zu kämpfen. Im Verlauf traten zunehmend therapieresistente Tumorschmerzen in den Vordergrund. Es wird beschrieben, wie wichtig es ist, die veränderte Schmerzqualität zu erkennen. Der Tumorschmerz mit neuropathischer Komponente beziehungsweise „*mixed pain*“ erschwerte im vorliegenden Fallbeispiel eine zufriedenstellende Symptomkontrolle. Es erfolgte ein Wechsel von transdermal appliziertem Fentanyl zu einem subkutanen Perfusorsystem mit Morphin und kurz darauf, aufgrund fehlender Schmerzkontrolle, auf ein orales retardiertes Oxycodonpräparat. Diese zweifache Opioidrotation wird im Folgenden diskutiert. Aspekte wie Resistenzentwicklung, inkomplette Kreuztoleranz und genetische Polymorphismen werden mithilfe wissenschaftlicher Literaturrecherche beleuchtet.

## Abstract (en)

This case report describes the situation of a 67-year-old chronic pain patient who is confronted with the diagnosis of metastatic small cell lung cancer due to an incidental finding. Previously, she had been struggling with chronic lumboischialgia. As she progressed, treatment-resistant tumor pain became increasingly prominent. The importance of recognizing the change in pain quality is described. The tumor pain with a neuropathic component or “mixed pain” made symptom control difficult in the present case report. A switch from transdermally applied fentanyl to a subcutaneous perfusor system with morphine was made and shortly thereafter, due to lack of pain control, to an oral sustained-release oxycodone preparation. This dual opioid rotation is discussed below. Aspects such as resistance development, incomplete cross-tolerance and genetic polymorphisms are highlighted with the help of scientific literature review.

## Einleitung

Das Auftreten einer metastasierten Tumorerkrankung verändert das Leben schlagartig. Mit der Diagnosestellung verändert sich häufig auch die Zuständigkeit der Behandlungsteams. Zuvor hat die Patientin die Schmerzambulanz regelmäßig aufgrund ihrer chronischen Wirbelsäulenbeschwerden aufgesucht. Es wurde eine stationäre Aufnahme auf einer internistischen Abteilung zur Behandlung der rezidivierend auftretenden Lumboischialgie erforderlich. Im weiteren Krankheitsverlauf veränderte sich das Schmerzbild und es traten zunehmend Tumorschmerzen im Bereich des linken Hemithorax in den Vordergrund.

Bei Vorhandensein von therapieresistenten Schmerzen ist es für das Behandlungsteam oft nicht leicht, die richtige Entscheidung zwischen Aufdosieren, Ergänzen oder Wechsel der Substanz zu treffen. In einer (wiederholten) gezielten Schmerzanamnese, körperlichen Untersuchung und Diagnostik kann häufig die Lösung zu einer nachfolgenden, individualisierten Therapieanpassung liegen. Leider blieben in der folgenden Falldarstellung nicht nur die Dosissteigerung von transdermalem Fentanyl, sondern auch die Rotation auf einen subkutanen Morphin-Perfusor erfolglos. Es wird augenscheinlich, dass eine Schmerztherapie nicht unbedingt besser wirksam sein muss, je invasiver sie ist. Unter Berücksichtigung des Vorhandenseins von neuropathischen Schmerzen bei der Patientin, wurde mit dem neuerlichen Substanzwechsel auf Oxycodon in oraler Form eine optimale Symptomkontrolle erreicht. In diesem Zusammenhang wird hier genauer auf die Begriffe „*mixed pain*“ sowie „*neuropathischer Tumorschmerz*“ und die Behandlungsmöglichkeiten eingegangen.

## Schilderung des Patientenbeispiels

### Teil 1 – Die chronische Schmerzpatientin mit malignem Zufallsbefund

Eine 67-jährige Patientin war aufgrund chronisch rezidivierender Lumboischialgien seit Jahren in der Schmerzambulanz in Behandlung. Eine vorbekannte Osteoporose mit Wirbelkörperfraktur, Infiltrationsbehandlungen und mehrere Wirbelsäulenoperationen fanden sich in der Langzeitanamnese. Die zu diesem Zeitpunkt etablierte Dauertherapie bestand aus einem transdermalen Fentanyl Pflaster (37 µg pro Stunde), sowie der oralen Gabe von 300 mg Gabapentin abends, 25 mg Pregabalin zweimal täglich und 10 mg Escitalopram morgens.

Im Rahmen der Abklärung eines intrapulmonalen Rundherdes wurde Anfang des Jahres 2018 ein kleinzelliges Bronchialkarzinom festgestellt. Bereits bei Diagnosestellung waren cerebrale Metastasen, Lebermetastasen und eine Expansion in der linken Nebenniere vorhanden.

Zu diesem Zeitpunkt war die Patientin in gutem Allgemeinzustand und normalem Ernährungszustand (Body-Mass-Index 24,5). Klinisch berichtete sie über vermehrte Müdigkeit, gelegentlichen Schwindel und von rezidivierenden Stürzen, sowie dem Vorliegen einer Harninkontinenz. Die Familienanamnese war positiv hinsichtlich einer Bronchuskarzinomerkrankung des Vaters sowie des Bruders. Das Risikoprofil ergab einen Nikotinabusus von vierzig pack years mit Abstinenz seit dreizehn Jahren. Eine tumorspezifische Therapie wurde seitens der Patientin wiederholt abgelehnt.

Nach einem weitgehend beschwerdearmen Sommer kam die Patientin Ende September in die internistische Ambulanz aufgrund einer zunehmenden Lumbalgie links mit, vor allem nächtlichen, Schmerzen im Lendenwirbelbereich ohne Ausstrahlung. Weiters gab sie Dyspnoe, produktiven Husten und Cephalea an. Die computertomographische Abklärung des Skeletts ergab hochgradig erosive Osteochondrosen in der Lendenwirbelsäule ohne Hinweis auf ossäre Metastasen. Die medikamentöse Therapie mit dem transdermalen Fentanyl Pflaster und der oralen Escitalopramgabe waren hinsichtlich der Dosierung unverändert geblieben. Sowohl Gabapentin als auch Pregabalin wurden in der Dokumentation hier nicht mehr erwähnt.

Es erfolgte die stationäre Aufnahme auf der internistischen Station und am nächsten Tag die Konsultation des Palliativkonsiliararztes. Dieser führte ein Anamnesegespräch mit der Patientin und erörterte die unterschiedlichen Möglichkeiten der Symptomkontrolle bei zunehmender Dyspnoe bis hin zur palliativen Sedierung. Weiters wurde die Steigerung des Fentanyl-Pflasters von 37 auf 50 µg pro Stunde empfohlen, sowie bei Auftreten von Schmerzspitzen oder Atemnot zu einer bedarfsweisen Einnahme von 2,6 mg Hydromorphon bis zu sechsmal täglich geraten.

Beim Folgebesuch drei Tage später berichtete die Patientin, dass sie die Steigerung des Fentanyl-Pflasters weder als positiv noch als negativ erlebt hätte, hingegen hätten die Paracetamol-Infusionen, die ergänzend verabreicht wurden, eine Schmerzlinderung gebracht. Nach einer Woche Aufenthalt konnten die Infusionen auf bedarfsweise Gaben von Paracetamol 500 mg Tabletten oder Metamizol Tropfen umgestellt und die Patientin in die häusliche Pflege entlassen werden.

### Teil 2 – Auftreten von Tumorschmerzen und Übernahme auf die Palliativstation

Ein Monat später erfolgte die stationäre Wiederaufnahme aufgrund einer Schmerzexazerbation und zunehmender Dyspnoe. Bei Aufnahme war die Patientin selbstständig mobil, jedoch in deutlich geschwächtem Allgemeinzustand. Sie gab starke Dauerschmerzen im linken Hemithorax, dem Bereich des Primärtumors, an und in der Auskultation fiel ein fehlendes Atemgeräusch links auf. Die medikamentöse Therapie war zu Hause wie folgt gesteigert worden: Fentanyl transdermales Pflaster 75 µg pro Stunde. Die Bedarfstherapie hatte sie aufgrund der starken Schmerzen ausgeschöpft: Hydromorphon 1,3 mg Kapseln benötigte sie zusätzlich sechsmal täglich. Weiters nahm sie selbstständig jeweils viermal täglich Paracetamol 500 mg Tabletten und 30 Tropfen Metamizol ein, auch wenn die zeitgleiche Einnahme dieser beiden zentral wirksamen Substanzen nicht vorgesehen war. Im Rahmen des Aufenthaltes kam es zu einer zunehmenden Kraftlosigkeit und Hypästhesie im Bereich der rechten oberen Extremität, sowie einer Schwäche des rechten Beines. Diese beginnende maligne Hemiparese rechts war aufgrund der größenprogredienten, frontoparietal links lokalisierten, cerebralen Metastasierung aufgetreten.

Die Umstellung der Schmerztherapie auf Paracetamol-Infusionen und bedarfsweise Morphin-Injektionen subkutan führte zu keiner Besserung. Es wurde daher, wie im eingeholten Palliativkonsil empfohlen, nach Entfernung des Fentanyl-Pflasters, ein Morphin-Perfusor subkutan etabliert. Initial wurde, bei einer errechneten Tagesäquivalenzdosis von 73 mg mit einer kontinuierlichen Laufrate von 48 mg Morphin begonnen. Die Tagesäquivalenzdosis errechnete sich folgendermaßen: 75 µg pro Stunde Fentanyl entsprechen 180 mg oralem Morphin. Ergänzend wurde die regelmäßig eingenommen Bedarfsmedikation von sechsmal täglich 1,3 mg Hydromorphon (= 39 mg Morphin per os) addiert. Insgesamt 219 mg orales Morphin werden durch drei dividiert und ergeben 73 mg Morphin subkutan. Anschließend an die sehr gering gewählte Startdosis erfolgte eine stufenweise Steigerung auf insgesamt 67,2 mg Tagesdosis Morphin subkutan. Anschließend wurde die Patientin auf die Palliativstation zur weiteren Symptomkontrolle übernommen.

Unter der laufenden Dosierung zeigte sich eine fehlende Schmerzlinderung. Vor allem bewegungsunabhängige Schmerzspitzen im Bereich des linken Hemithorax waren belastend für sie. Die Patientin war äußerst unzufrieden mit der eingeschränkten Mobilität durch die Perfusorvorrichtung. Im Gespräch mit der Patientin wurde nun die Rückumstellung auf ein orales Präparat und eine weitere Opioidrotation besprochen. 67 mg Morphin subkutan entsprechen, mit drei multipliziert, 201 mg oralem Morphin. Orales Morphin ist doppelt so schwach wie Oxycodon, daher ergeben sich daraus 100 mg orales Oxycodon. Die errechnete Äquivalenzdosis wurde aufgrund der Rotation um ein Drittel reduziert und es wurde mit Oxycodon 40 mg retard Tabletten zweimal täglich begonnen. Die Paracetamol-Infusionen wurden vorerst weitergeführt und Metamizol konnte auf 30 Tropfen abends reduziert werden. Zusätzlich wurde die Bedarfsmedikation mit Oxynorm 10 mg Tabletten maximal sechsmal täglich ergänzt. Als antiödematöse Therapie hinsichtlich der symptomatischen cerebralen Metastasierung wurde einmal täglich morgens Dexamethason 20 mg intravenös verabreicht.

In weiterer Folge war eine minimale Steigerung von Oxycodon auf 45 mg retard Tabletten zweimal täglich notwendig, worunter eine optimale Schmerzkontrolle erreicht und die Paracetamol-Infusionen sogar abgesetzt werden konnten. Die neurologische Symptomatik und Cephalgie besserten sich deutlich und Dexamethason konnte auf orale Verabreichung und mittels stufenweiser Reduktion auf die kleinstmögliche Erhaltungsdosis von 6 mg Tabletten umgestellt werden. Nach diesem zweiwöchigen Aufenthalt konnte die Patientin nach Hause entlassen werden, wo sie durch eine Freundin unterstützt, selbstständig versorgt war.

Häusliche Stürze mit Frakturen der Clavicula und des Schambeins waren Gründe für zwei weitere stationäre Aufenthalte im November, wobei dennoch die laufende Schmerztherapie völlig unverändert blieb. Die Patientin wurde jeweils wieder in die häusliche Umgebung entlassen. Ende November erfolgte die Wiederaufnahme auf der Palliativstation aufgrund zunehmender Immobilität und Verwirrtheit. Drei Tage später verstarb sie dann doch unerwartet plötzlich unter dem klinischen Verdacht des Vorliegens einer Pulmonalembolie.

## Fragestellung

Welche medikamentösen Therapieoptionen können in Zusammenhang mit chronisch degenerativen Wirbelsäulenbeschwerden und progredientem Tumorschmerz im palliativen Setting gefunden werden?

Welche schmerztherapeutischen Limitationen können für transdermal appliziertes Fentanyl und die subkutane kontinuierliche Morphinverabreichung benannt werden?

Welche pharmakologischen Faktoren sind bei Durchführung einer Opioidrotation zu beachten?

## Diskussion

### Einleitung

Im vorliegenden Fallbeispiel fällt auf, wie flexibel das Behandlungsteam in Therapieentscheidungen bleiben muss, um eine zufriedenstellende Symptomkontrolle erreichen zu können. Die Situation der Patientin mit chronischen Wirbelsäulenbeschwerden änderte sich durch Diagnosestellung des metastasierten Bronchialkarzinoms schlagartig. Im Laufe der Patientenbetreuung zeigte sich, wie rasch sich die Schmerzätiologie ändern kann und wie wichtig es aus schmerztherapeutischer Sicht ist, streng schrittweise und nach Indikation vorzugehen, um nicht Gefahr zu laufen, mit der Patientin einen falschen Behandlungspfad einzuschlagen. Hierbei kann ein Tool wie die „SOP – Schmerztherapie bei Palliativpatienten“ von Viehrig M. et al. hilfreich sein [1].

Eine strenge Prüfung der Indikation beziehungsweise eine klare Diagnosestellung muss der Entscheidung zur passenden Therapie vorausgehen, um eine Symptomlinderung zu erreichen. Ein schrittweises Vorgehen ist dabei unerlässlich und sollte niemals die Schritte der Anamnese und der körperlichen Untersuchung überspringen. Selbst bei Vorliegen einer metastasierten Tumorerkrankung ist eine leitliniengerechte Behandlung der gleichzeitig vorhandenen degenerativen Veränderungen an der Wirbelsäule unerlässlich. Im Rahmen der Fallrecherche war auffällig, dass die ausführlichste Dokumentation über den Schmerz in den physiotherapeutischen Befunden zu finden war. Diese Ausführungen betrafen nicht nur die Schmerzlokalisation, die Schmerzausstrahlung und die Qualität des Schmerzes, sondern berücksichtigten auch das zeitliche Auftreten desselbigen und umfassten sogar den Schmerz verstärkende oder lindernde Faktoren.

In der Leitlinie für das Management akuter, subakuter, chronischer und rezidivierender unspezifischer Kreuzschmerzen 2018 [2] finden sich sehr genaue Empfehlungen zur medikamentösen Schmerztherapie, wobei zu erwähnen ist, dass zuvor alle nicht-medikamentösen Maßnahmen ausgeschöpft werden sollten. Eine Opiattherapie in Erwägung gezogen werden, allerdings ist aufgrund der schlechten transdermalen Dosierungsmöglichkeit eine orale Applikation vorzuziehen [2, S. 62 f].

### Tumorschmerz und „*mixed pain*“

Im zweiten Teil des Fallberichts kam es bei der Patientin zu einer stationären Wiederaufnahme aufgrund deutlicher Befundprogredienz und einer damit verbundenen Schmerzeskalation im linken Hemithorax. Ein malignes Schmerzgeschehen war nun vorherrschend, das in erster Linie als „*mixed pain*“ eingeordnet werden muss. Durch die einwachsende und verdrängende Tumormasse entsteht eine Mischung aus nozizeptivem und neuropathischem Schmerz [3, 4, S. 617].

In den Neuerungen der WHO-Guidelines zum Thema Tumorschmerz wird deutlich eine patientenorientierte Strategie in den Fokus gestellt. Die Entscheidung zur passenden Opiattherapie soll nach individuellen Bedürfnissen angepasst werden [5].

Die Anwendung von Stufe III-Opioiden wird bei einer hohen Schmerzintensität in den S3-Leitlinien für Palliativmedizin empfohlen [6]. Morphin, Hydromorphon und Oxycodon sind dabei Präparate der ersten Wahl [6, S. 166 f]. In einer Studie von Corli et al. wurden orales Morphin, orales Hydromorphon, transdermales Buprenorphin und Fentanyl miteinander verglichen [7]. Bei annähernd gleicher Wirksamkeit, in Bezug auf die Abnahme der Schmerzintensität, musste das transdermale Fentanyl häufiger gesteigert werden [7].

### Opioidrotation von transdermaler Fentanyl-Applikation zur subkutanen kontinuierlichen Morphinverabreichung

Die Sinnhaftigkeit des Wechsels von Fentanyl auf ein anderes starkes Opioid lässt sich pharmakologisch mit dem Phänomen der „inkompletten Kreuztoleranz“ begründen [8]. Das bedeutet, dass die Wirkweise der einzelnen Opioide, vermutlich genetisch bedingt, sehr unterschiedlich sein kann. Deshalb kann die Rotation innerhalb der Substanzgruppe zu einer verbesserten Wirkung oder zu einer Reduktion der Nebenwirkungen bei äquipotenter Dosierung führen.

Auch eine Veränderung des Applikationsweges kann sinnvoll sein, denn transdermale Systeme sind zur Behandlung einer Schmerzeskalation ungeeignet, da es nur langsam zum Erreichen eines adäquaten Wirkstoffspiegels kommt (frühestens 12–24 h nach Applikation). Sie eignen sich daher streng genommen nur zur Behandlung eines stabilen Schmerzniveaus [9]. Beim Wechsel von einem Opioid-Pflaster zu einer anderen Applikationsform ist außerdem die lange Halbwertszeit des Opioids nach Entfernung des Pflasters zu beachten [8]. Eine Kachexie, die aufgrund des verminderten subkutanen Fettgewebes zur reduzierten Wirkstoffaufnahme über die Haut führt, sollte ebenfalls berücksichtigt werden [9].

Weitere Gründe für eine Opioidrotation können bei Vorliegen einer fehlenden Schmerzlinderung trotz adäquater Dosissteigerung, das Auftreten von unerwünschten Wirkungen sein, wie beispielsweise Somnolenz, therapieresistenter Übelkeit oder Obstipation [8]. Allerdings können auch genetische Faktoren eine Rolle spielen, wenn eine schlechte analgetische Wirksamkeit bei der Behandlung von tumorbedingten Schmerzen mit Opioiden beobachtet wird [10, S. 15]. Genetische Polymorphismen sind weiters ein wichtiger patientenindividueller Faktor in Bezug auf Enzymsysteme. Für „*poor Metabolizer*“ und „*ultra-rapid Metabolizer*“ ergeben sich unterschiedliche Interaktionspotentiale. Arzneimittelwechselwirkungen kommen durch Induktion oder Inhibition des Cytochrom-P450-Enzymsystems in der Leber zustande. Beispielsweise ist Fentanyl Substrat von CYP3A4 und seine Wirksamkeit und Wirkdauer kann durch Enzyminduktoren wie Carbamazepin, Phenytoin, Dexamethason, Johanniskraut oder Rifampicin vermindert werden [11, S. 51].

**Figure 1:**
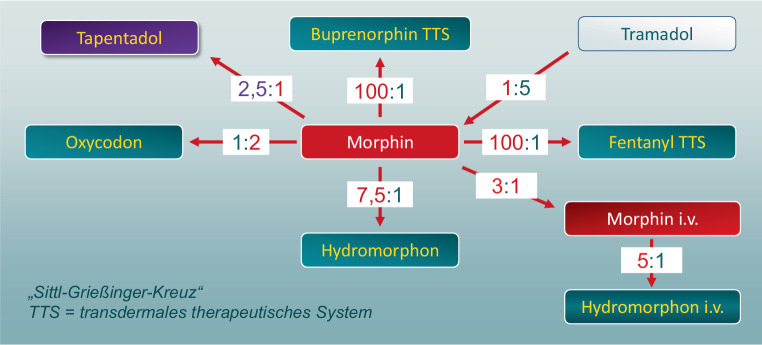

Für den Wechsel von einem Opioid zu einem anderen werden sogenannte Äquipotenztabellen für die Umrechnung der Dosierung herangezogen, die hauptsächlich auf Erfahrungswerten beruhen [8]. Ein Beispiel hierfür findet sich in Abb. 1.

Bei Wechsel aufgrund nicht ausreichender Schmerzlinderung kann die äquipotente Dosis verwendet werden. Bei einer notwendigen Opioidrotation aufgrund von Nebenwirkungen, muss die errechnete äquipotente Dosierung um ein Drittel reduziert werden, um zur Anfangsdosis des neuen Opioids zu gelangen. Anschließend erfolgt die stufenweise Titration bis zur ausreichenden Symptomlinderung [6, S. 176]. Dies gilt vor allem bei Verdacht auf eine partielle Opioidresistenzentwicklung und bei der Umstellung von einer transdermalen auf eine andere Applikationsform aufgrund der langen Halbwertszeit des Opioids nach Pflasterentfernung [9]. Beim Wechsel von einer oralen zu einer subkutanen Applikationsform von Morphin ist die Dosierung auf ein Drittel bis zur Hälfte zu reduzieren [6]. Die subkutane kontinuierliche Verabreichung von Morphin ist eine mögliche invasive Darreichungsform, ebenso wie die Verabreichung über Porth-a-Cath-Systeme oder PICCs (Peripherally Inserted Central Venous Catheter). Sie finden Anwendung bei Vorliegen von therapieresistenten Schmerzen und schlechtem Allgemeinzustand. Die Titration bei der subkutanen Anwendung kann, ähnlich wie bei der parenteralen Gabe, zügig erfolgen. Ein weiterer Vorteil der kontinuierlichen subkutanen Gabe ist die gute Steuerbarkeit der Dosierung. Ein Nachteil dieser Art der Schmerzmittelzufuhr ist die notwendige subkutane Leitung mit dem angehängten Perfusorsystem, was im Speziellen bei starker motorischer Unruhe hinderlich sein kann.

### Wechsel von subkutaner kontinuierlicher Morphinverabreichung zu Oxycodon retard Tabletten

Die Kehrtwende im beschriebenen Fallbeispiel stellt die zweite Rotation innerhalb der Gruppe der starken Opioide und gleichzeitig der erneute Wechsel der Applikationsform zurück auf eine orale Verabreichung dar. Erwähnenswert ist der Umstand, dass zum Zeitpunkt der neuerlichen Opioidrotation die äquivalente Tagesdosis des subkutanen Morphins im Vergleich zum transdermalen Fentanyl noch gar nicht erreicht worden ist. Abgesehen von dieser Tatsache lagen auch keine Gründe für eine parenterale Schmerzmittelgabe vor, denn die Patientin litt weder unter unstillbarem Erbrechen oder einer Schluckstörung, noch bestand eine massive allgemeine Schwäche, die diesen Verabreichungsweg erfordern würde [6, S. 173]. Aufgrund des Leidensdrucks und der unzureichenden Schmerzkontrolle unter dem kontinuierlich subkutan verabreichten Morphin, wurde diese erneute Umstellung im beschriebenen Fall vollzogen. Bei Vorliegen einer neuropathischen Schmerzkomponente wird aus der Gruppe der niedrigpotenten Opioide Tramadol und bei den starken Opioiden Buprenorphin und Oxycodon eine verbesserte Wirksamkeit zugeschrieben [10, S. 16, 13, S. 75]. Das Vorhandensein des „*mixed pain*“ und der Patientenwunsch nach einer oralen Applikationsform lieferten in diesem Fall die Entscheidungsgrundlage für die Umstellung auf eine orale retardierte Verabreichung von Oxycodon mit entsprechender Bedarfsmedikation.

Bei schlechtem Ansprechen auf Opioide sollte in der Behandlung von Tumorschmerzen mit neuropathischem Charakter die Ergänzung von adjuvanten Analgetika erwogen werden, auch wenn in der Kombination häufiger unerwünschte Wirkungen wie Müdigkeit und Schwindel auftreten [6, S. 190].

Die hinzugekommene Hypästhesie der rechten Körperhälfte mit beginnender Hemiparese rechts ist als Minussymptomatik eines neuropathischen Schmerzes zu bewerten [6, S. 162]. Die neurologische Symptomatik aufgrund der progredienten intrakraniellen Metastasierung konnte mithilfe der antiödematösen Wirkung von Dexamethason verbessert werden [14]. Die entzündungshemmende Eigenschaft von Cortison wirkte sich schmerzmodulierend aus und so konnte schließlich eine deutlich vereinfachte und zufriedenstellende Schmerztherapie für die Patientin gefunden werden.

## Conclusio

Das Vorliegen von therapieresistenten Schmerzen ist für die Betroffenen äußerst qualvoll und stellt auch die behandelnden Personen vor große Herausforderungen. In solchen Situationen ist es unabdingbar die Anamneseerhebung zur vertiefen oder sogar neu auszurollen, sowie die laufende Therapie kritisch zu hinterfragen. Ganz besonderes Augenmerk sollte auf die Erhebung der Schmerzentität, insbesondere dem Vorhandensein einer neuropathischen Komponente, gelegt werden, um den korrekten Behandlungspfad einschlagen zu können. Eine Opioidrotation bei therapieresistenten Tumorschmerzen kann mit der nötigen Erfahrung und einer Kombination aus palliativmedizinischem und schmerztherapeutischem Wissen sehr erfolgreich sein. Erstaunlich ist, wie niedrig die ausreichende Dosierung, nach dem Wechsel zur passenden Substanz, sein kann, was den Vorteil geringerer Nebenwirkungen mit sich bringt.
